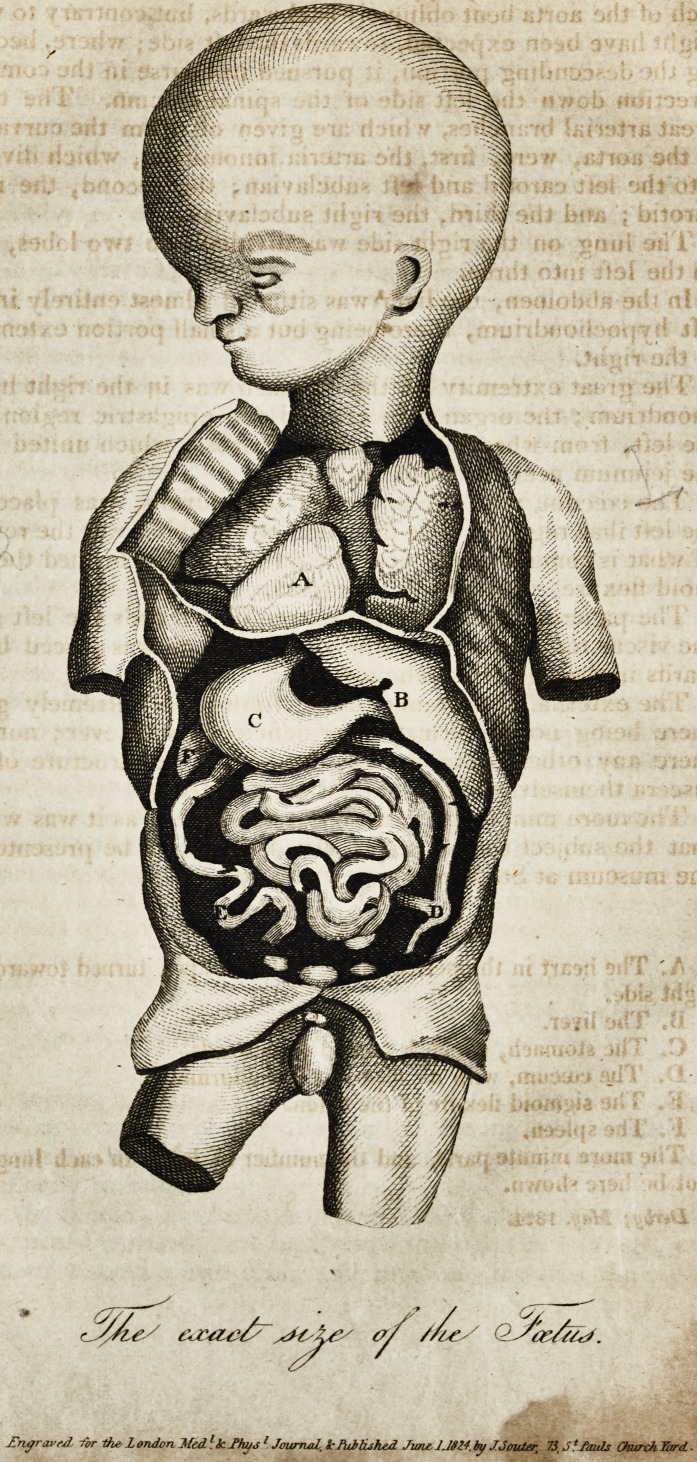# A Case of the Reversed Position of the Thoracic and Abdominal Viscera

**Published:** 1824-06

**Authors:** Douglas Fox

**Affiliations:** Member of the Royal College of Surgeons, London.


Art. IV.—*A Case of the reversed Position of the Thoracic and Abdominal Viscera.* By Douglas Fox, Member of the Royal College of Surgeons, London.

[With an Engraving.]

In dissecting a fœtus, expelled about the fifth month of pregnancy, in consequence of the mother having inflammation of the bowels, the thoracic and abdominal viscera were found completely reversed.

In the thorax, the heart was seen placed obliquely, with the basis turned upwards and backwards towards the left side, and the apex turned downwards and forwards towards the right,

475

*The exact size of the Fœtus.*

Engraved for the London Med[l]. & Phys[l]. Journal, & Published June 1. 1824. by J. Souter, 73, S[t]. Pauls Church Yard.

being situated exactly opposite to what is usual. The transverse arch of the aorta bent obliquely backwards, but contrary to what might have been expected, towards the left side; where, becoming the descending portion, it pursued its course in the common direction down the left side of the spinal column. The three great arterial branches, which are given off from the curvature of the aorta, were, first, the arteria innominata, which divided into the left carotid and left subclavian; the second, the right carotid; and the third, the right subclavian.

The lung on the right side was divided into two lobes, that on the left into three.

In the abdomen, the liver was situated almost entirely in the left hypochondrium, there being but a small portion extending to the right.

The great extremity of the stomach was in the right hypochondrium; the organ then crossed the epigastric region into the left, from whence the duodenum arose, which united with the jejunum near the right kidney.

The cœcum, with the appendix vermiformis, was placed in the left iliac region. The direction of the colon was the reverse of what is common, and near the termination it formed the sigmoid flexure, in the right iliac fossa.

The pancreas had the great extremity towards the left side; the viscus terminated near the spleen, which was placed backwards in the right hypochondrium.

The external appearance of the fœtus was extremely good, there being no malformation or deficiency whatever; nor was there any other irregularity in the shape or structure of the viscera themselves than what has been stated.

The more minute parts were not examined, as it was wished that the subject should be preserved entire, to be presented to the museum at St. Bartholomew's Hospital.

### *Description of the Plate.*

A. The heart in the pericardium, with the apex turned towards the right side.

B. The liver.

C. The stomach, which is turned downwards.

D. The cœcum, with its appendix vermiformis.

E. The sigmoid flexure of the colon.

F. The spleen.

The more minute parts, and the number of lobes in each lung, cannot be here shown.

*Derby; May,* 1824.

## Figures and Tables

**Figure f1:**